# Genome-wide association study of yield and yield-related traits in pepper (*Capsicum annuum* L.)

**DOI:** 10.3389/fpls.2026.1926954

**Published:** 2026-10-22

**Authors:** Xiaocui Chen, Xiaowei Zhang, Shichao Sun, Hong Yang, Cheng Qin, Dan Zhou, Di Peng, Qingqing Li, Xiaosong Gou, Ao Wang

**Affiliations:** 1 Zunyi Academy of Agricultural Sciences, Zunyi, China; 2 State Key Laboratory of Tropical Crop Breeding, Shenzhen Branch, Guangdong Laboratory of Lingnan Modern Agriculture, Key Laboratory of Synthetic Biology, Ministry of Agriculture and Rural Affairs, Agricultural Genomics Institute at Shenzhen, Chinese Academy of Agricultural Sciences, Shenzhen, China; 3 Guizhou Academy of Agricultural Sciences, Guiyang, China

**Keywords:** fruit architecture, genome-wide association study (GWA), Guizhou germplasm, linkage disequilibrium, SLAF-seq

## Abstract

Fruit morphology and yield are major determinants of pepper market value and productivity, but the genetic basis of these traits remains incompletely resolved. In this study, we evaluated 195 Capsicum annuum accessions for six fruit morphology and yield-related traits in two environments. The traits showed substantial variation, with coefficients of variation of 40.43-57.09% and broad-sense heritability of 0.59-0.97. Reduced-representation sequencing generated 76,418 high-quality single nucleotide polymorphisms, and population analyses resolved eight genetic subgroups. Genome-wide association analysis detected 23 significant loci, comprising 12 loci for fruit morphology and 11 loci for yield-related traits. These loci contained 318 unique genes. Notably, the major fruit-length loci *qFL2* and *qFL5* contained gibberellin 20 oxidase 1 (*GA20ox1*) and an OVATE family protein gene corresponding to the functionally characterized pepper fruit-elongation regulator *CaOFP20*, respectively. These loci, allele-trait associations, and putative candidate genes provide a basis for further validation and marker development in pepper breeding.

## 1 Introduction

Pepper (*Capsicum annuum L.*) is one of the most widely cultivated vegetable crops globally and represents a major source of dietary nutrients, flavor compounds, and bioactive metabolites (Parisi et al., 2020). Among the traits that determine its commercial and agronomic value, fruit morphology and yield-related characteristics are of particular importance, as they directly influence consumer preference, processing suitability, and overall productivity. Improving these traits has therefore been a central objective in pepper breeding programs (Lahbib et al., 2023; Wu et al., 2019).

China is a center of diversity and production for cultivated pepper, with several regions harboring rich and underexploited genetic resources (Zhang et al., 2016). In particular, Guizhou Province is recognized as a key pepper-producing area with diverse agro-ecological conditions that have contributed to the accumulation of substantial phenotypic and genetic variation in local landraces and cultivars (Zou and Zou, 2021). However, systematic evaluation of this germplasm, especially at the genomic level, remains limited, and its potential for uncovering novel loci associated with agronomically important traits has not been fully realized.

Previous mapping studies have established that pepper fruit morphology and yield are quantitatively inherited, but their resolution and transferability remain uneven. Biparental QTL studies can detect large-effect loci within controlled crosses, yet they sample only the alleles segregating between two parents. Conversely, association studies survey broader diversity but are sensitive to population structure and often stop at statistical loci without defining LD-supported intervals or evaluating biologically plausible genes. Consequently, loci reported in one genetic background may not explain variation in locally adapted Guizhou germplasm, and the functional interpretation of many association signals remains incomplete (Rao et al., 2003; Han et al., 2016; Colonna et al., 2019; Nimmakayala et al., 2016; Lee et al., 2020).

Reduced-representation sequencing approaches, including specific-locus amplified fragment sequencing (SLAF-seq), provide dense genome-wide markers at comparatively low cost and are therefore well suited to genetically diverse crop panels (Wu et al., 2019). When population structure, relatedness, and genome-wide linkage disequilibrium (LD) are explicitly considered, GWAS can connect natural variation with bounded candidate intervals rather than isolated significant SNPs. This combined framework is especially useful for undercharacterized regional germplasm, in which both novel alleles and locally enriched haplotypes may be present.

In this study, we assembled a diverse panel of 195 *C. annuum* accessions collected from Guizhou Province and evaluated six fruit morphology and yield-related traits across two environments. Using SLAF-seq-derived SNP markers, we characterized the genetic diversity and population structure of this germplasm collection and performed GWAS to identify loci associated with key agronomic traits. We further evaluated phenotypic differences between allelic classes and prioritized putative candidate genes within LD-supported intervals. Collectively, this work aims to enhance our understanding of the genetic architecture underlying fruit morphology and yield in pepper and to provide valuable genomic resources for future breeding efforts.

## 2 Materials and methods

### 2.1 Plant materials and field trials

A total of 195 pepper accessions, including local germplasm resources and stable breeding lines from Guizhou Province, China, were used in this study. These materials were provided by the Yang Hong research group at the Pepper Research Institute, Guizhou Academy of Agricultural Sciences. Field experiments were conducted in 2020 and 2021 at the experimental station of the Pepper Research Institute, Guizhou Academy of Agricultural Sciences, located in Xinpu Town, Honghuagang District, Zunyi City, Guizhou Province, China (27°43′28″ N, 107°02′54″ E; altitude 840 m).

The experiment was arranged in a randomized complete block design with three biological replicates. Each accession was planted in two rows with 15 plants per row (30 plants per plot), using a row spacing of 60 cm and a plant spacing of 25 cm. Standard pepper cultivation and field management practices were applied throughout the growing season.

Phenotypic evaluation was conducted at the mature fruit stage using 10 representative plants from the internal positions of the two rows after excluding the first and last plants of each row. Six quantitative traits were investigated, including fruit length (Fl), fruit transverse diameter (Ftd), fruit flesh thickness (Fft), fruit number per plant (Fnp), single fruit weight (Sfw), and yield per plant (Yld). Fl, Ftd, and Fft were measured using a digital caliper. Sfw was determined using an electronic balance, and Fnp was counted manually. Yld was calculated as the total fresh weight of pepper fruits from three successive harvests. Mean values of the 10 sampled plants were used for subsequent analyses.

### 2.2 SLAF-seq library preparation, read alignment and SNP detection

#### 2.2.1 Library preparation and sequencing

Approximately 100 mg of young leaf tissue was collected from each accession at the early fruiting stage, immediately frozen in liquid nitrogen, and stored at −80 °C until DNA extraction. Genomic DNA was extracted using the CTAB method (Siddique et al., 2019; Solomon et al., 2019). Samples with DNA concentrations ≥20 ng/µL and volumes ≥30 µL were used for SLAF-seq library preparation. Libraries were sequenced with paired-end reads on the Illumina HiSeq platform. Reads containing adapter sequences were removed; read pairs were discarded when either read contained >10% ambiguous bases (N).

#### 2.2.2 Read alignment, SNP calling and filtering

Clean reads were aligned to the Capsicum reference genome (Zunla-1, version 2.0; https://solgenomics.net/ftp/genomes/Capsicum_annuum/C.annuum_zunla/assemblies/) using BWA-MEM v0.7.10-r789 (Li and Durbin, 2009; command: bwa mem -t 4 -M), and the alignments were sorted with SAMtools v1.9 (Li et al., 2009). SNPs were called independently with GATK v3.8 UnifiedGenotyper (McKenna et al., 2010) and SAMtools v1.9; only SNPs detected by both methods were retained. An initial filter of minor allele frequency (MAF) >0.01 and site integrity (INT; proportion of samples with nonmissing genotypes) >0.10 was applied. A stricter filter of MAF >0.05 and INT >0.80 yielded 76,418 SNPs for downstream analyses. The SLAF tag depth in **Supplementary Table S1** describes the mean depth of recovered restriction fragments per sample, it is not whole-genome sequencing coverage.

### 2.3 Population structure and phylogenetic analyses

Population structure and genetic relationships among individuals were investigated using principal component analysis (PCA), model-based clustering, and phylogenetic reconstruction. PCA was performed using PLINK v1.9 (Purcell et al., 2007) based on a pruned set of SNPs to reduce linkage disequilibrium bias (independent SNPs were obtained using the command --indep-pairwise 50 5 0.2). The first two principal components were extracted and used to visualize genetic variation and clustering patterns among individuals. Population structure was further inferred using ADMIXTURE v1.3.0 (Alexander et al., 2009), with the same LD-pruned dataset. A range of ancestral population numbers (K values) was tested, and each K was run with multiple replicates using different random seeds. The optimal K was determined based on the lowest cross-validation error, and individual ancestry proportions were visualized accordingly. In addition, a phylogenetic tree was constructed using SNP data derived from VCF files. Genetic distances were calculated and a tree was generated using VCF2is (Xu et al., 2025), and the resulting phylogeny was visualized and annotated using iTOL (Interactive Tree Of Life) (Letunic and Bork, 2024), with population information and ADMIXTURE results mapped onto the tree to facilitate interpretation of genetic relationships.

### 2.4 Genome-wide association study

GWAS for the six traits were conducted using the Fixed and Random Model Circulating Probability Unification (FarmCPU) algorithm implemented in the R package rMVP (Yin et al., 2021). To effectively control for false positives while maintaining statistical power, population structure was adjusted by incorporating the top three principal components as covariates, and cryptic relatedness among accessions was corrected using a kinship matrix calculated via the VanRaden method within rMVP. The genome-wide significance threshold was strictly determined using a modified Bonferroni correction defined as *P* = 0.01/*N*, where *N* represents the total number of high-quality SNPs analyzed. The genetic effects of these significant SNPs were assessed by evaluating the phenotypic differences between individuals with contrasting genotypes at each SNP. SNPs that had no significant impact on the corresponding traits were subsequently excluded. The genomic distributions of significant SNPs were visualized via Manhattan plots generated directly through the CMplot package (Yin et al., 2021). The percentage of phenotypic variance explained (PVE) by each lead SNP was estimated as *β*^2^/(*β*^2^+*NSE*^2^)×100, where *β* is the estimated allelic effect, *SE* is its standard error, and *N* is the number of accessions included in the GWAS.

### 2.5 Linkage disequilibrium decay and candidate-gene annotation

Genome-wide LD decay was estimated for the 195-accession association panel using PopLDdecay v3.43 (Zhang et al., 2019). Pairwise squared allele-frequency correlations (*r*^2^) were calculated from the quality-filtered VCF with a maximum intermarker distance of 2,000 kb, a minor allele frequency threshold of 0.05, and a maximum missing-data rate of 0.20. Of the 76,418 SNPs retained after upstream quality control, 76,368 passed the PopLDdecay filters. Mean *r*^2^ values were summarized by physical-distance bins, and a LOESS curve (span = 0.10) weighted by the number of SNP pairs in each bin was fitted to the decay profile. LD half-decay was defined as the distance at which fitted *r*^2^ declined to half of the maximum mean *r*^2^ observed within 1 kb. The exact crossing was 414 kb, with a sensitivity range of 414–430 kb across LOESS spans of 0.05-0.20; a rounded distance of 420 kb was therefore used as the interval radius.

Candidate genes within the GWAS-associated intervals were identified using the Zunla-1 reference genome annotation (version 2.0). For each lead SNP, a candidate interval extending 420 kb upstream and downstream was defined according to the estimated genome-wide LD decay distance. Gene coordinates, gene IDs, genomic locations, and functional descriptions were obtained from the corresponding NCBI gene models.

## 3 Results

### 3.1 Population structure and phylogenetic relationships of the Guizhou pepper germplasm

A total of 195 pepper accessions were collected from Guizhou Province. SLAF-seq generated 367,285,237 clean reads in these accessions (367.29 million reads; mean 1.88 million per accession), with mean Q30 and GC contents of 93.23% and 39.89%, respectively. The average depth of recovered SLAF tags was 12.31× per accession (**Supplementary Table S1**). Bioinformatic analysis identified 889,866 distinct SLAF tags across the panel, of which 285,337 were polymorphic, and 1,468,645 SNP sites were initially identified. After filtering, 76,418 high-quality SNP markers were retained for subsequent analyses. These markers were distributed across all 12 pepper chromosomes (**Supplementary Figure S1**) and were used for population genetic analyses and genome-wide association studies.

To investigate the genetic relationships among the 195 pepper accessions, population structure analysis was performed using ADMIXTURE with the number of ancestral populations (K) ranging from 1 to 10. Cross-validation error analysis indicated that the optimal population number was K = 8 (**Supplementary Figure S2**). Based on the ancestry coefficients, the entire population could be divided into 8 distinct subgroups (**Figure 1A**). Principal component analysis (PCA) further supported the population structure results. The first three principal components explained 33.30%, 14.40% and 8.38% of the total genetic variation, respectively (**Figure 1B**). Accessions belonging to the same subgroup tended to cluster together, whereas several admixed accessions were distributed between subgroups, indicating mixed ancestry within the collection. A neighbor-joining (NJ) phylogenetic tree was subsequently constructed based on genome-wide SNP data. The phylogenetic relationships were largely consistent with the ADMIXTURE and PCA results, with most accessions grouped according to their inferred population structure (**Figure 1C**). However, several accessions showed mixed clustering patterns, reflecting the complex breeding history and genetic background of Guizhou pepper resources.

**Figure 1:**
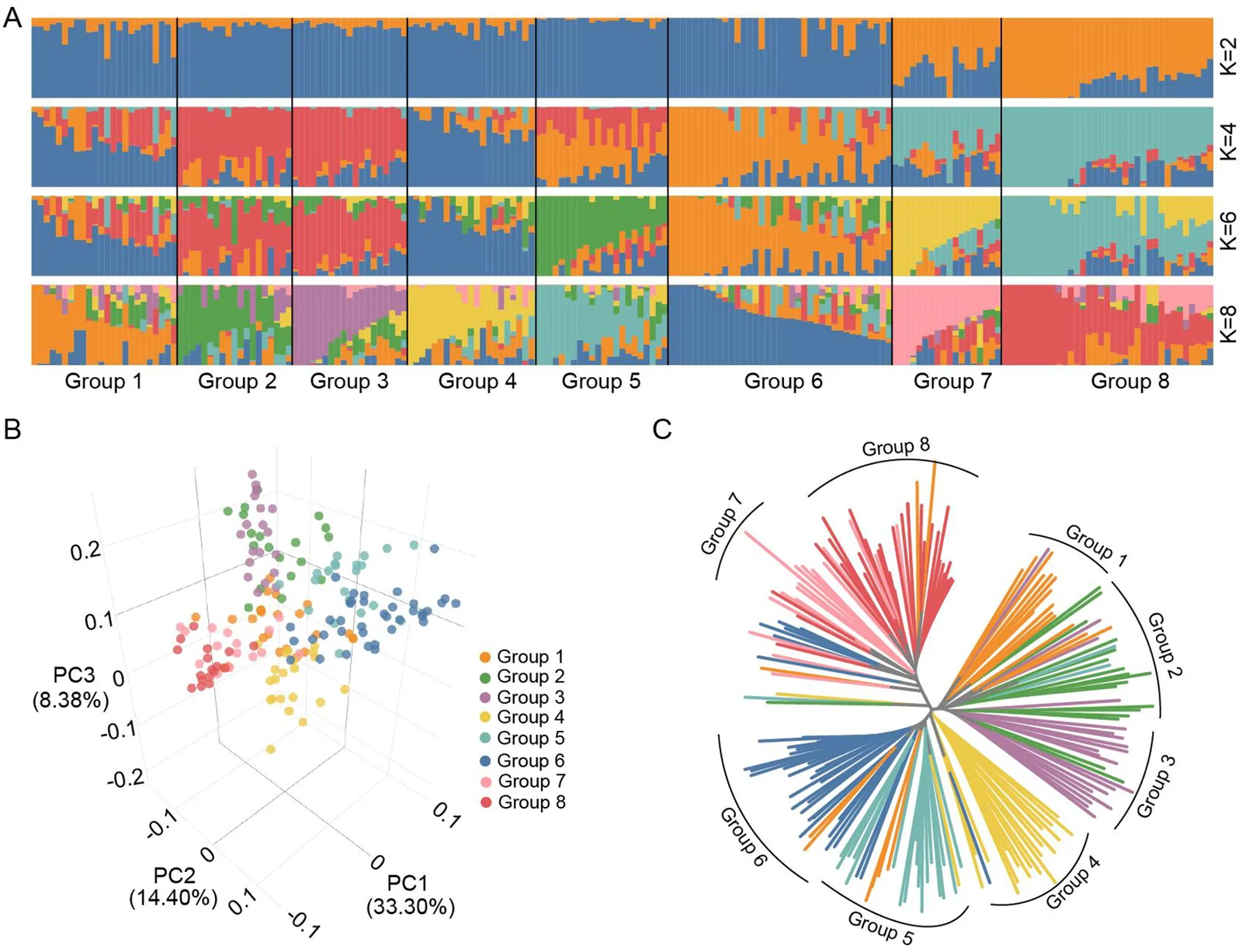
Population structure, principal component analysis, and phylogenetic relationships of 195 pepper germplasm accessions based on SNP markers. **(A)** Population structure analysis of 195 pepper accessions at K = 2, 4, 6, and 8. Each vertical bar represents an individual accession, and different colors indicate the estimated membership proportions in each genetic cluster. The accessions were classified into eight groups (Groups 1–8). **(B)** Three-dimensional principal component analysis (PCA) based on genome-wide SNP markers. Each point represents an individual accession, and colors correspond to the eight inferred groups. The percentages shown on the axes indicate the proportion of genetic variation explained by each principal component. **(C)** Neighbor-joining phylogenetic tree constructed using high-quality SNP markers. Different colors represent the eight genetic groups identified in the population structure analysis.

### 3.2 Phenotypic variation of fruit morphology and yield traits

To assess variation of fruit morphology and yield traits in this collection, these 195 pepper accessions were planted during two consecutive years and evaluated for six agronomic traits. Best linear unbiased prediction (BLUP) values were calculated to reduce environmental effects and improve phenotypic accuracy. All traits exhibited substantial phenotypic variation (CV = 40.43–57.09%), with fruit length (FL, 57.09%), fruit number per plant (Fnp, 55.81%), and fruit flesh thickness (Fft, 55.27%) showing the highest variability, indicating considerable breeding potential for fruit morphology and yield components (**Table 1**).

**Table 1:** Survey and statistics of yield and related traits in capsicum.

Trait	Mean	Min	Max	SD	CV (%)
Fnp	76.54	19.00	292.00	42.72	55.81
FL	6.44	1.60	26.30	3.68	57.09
Ftd	1.94	0.80	4.10	0.81	41.64
Fft	0.19	0.04	1.58	0.10	55.27
Sfw	8.25	1.64	24.82	4.14	50.12
Yld	1173.13	306.32	2825.68	474.29	40.43

These traits displayed continuous and approximately normal distribution, consistent with quantitative inheritance (**Figure 2**). Correlation analysis revealed significant associations among these traits. Notably, yield (Yld) was positively correlated with single fruit weight (Sfw, *r* = 0.44, *P* = 1.59e-10), Fnp (*r* = 0.24, *P* = 7.76e-04), Fft (r = 0.33, *P* = 1.83e-06), and fruit transverse diameter (Ftd, *r* = 0.29, *P* = 3.45e-05), but not with FL (*r* = 0.02, *P* = 0.78). Fruit length and diameter were strongly correlated (*r* = -0.64, *P* = 1.32e-23) and moderately correlated with Fft (*r* = -0.51, *P* = 2.94e-23), indicating coordinated variation among fruit morphology traits.

**Figure 2:**
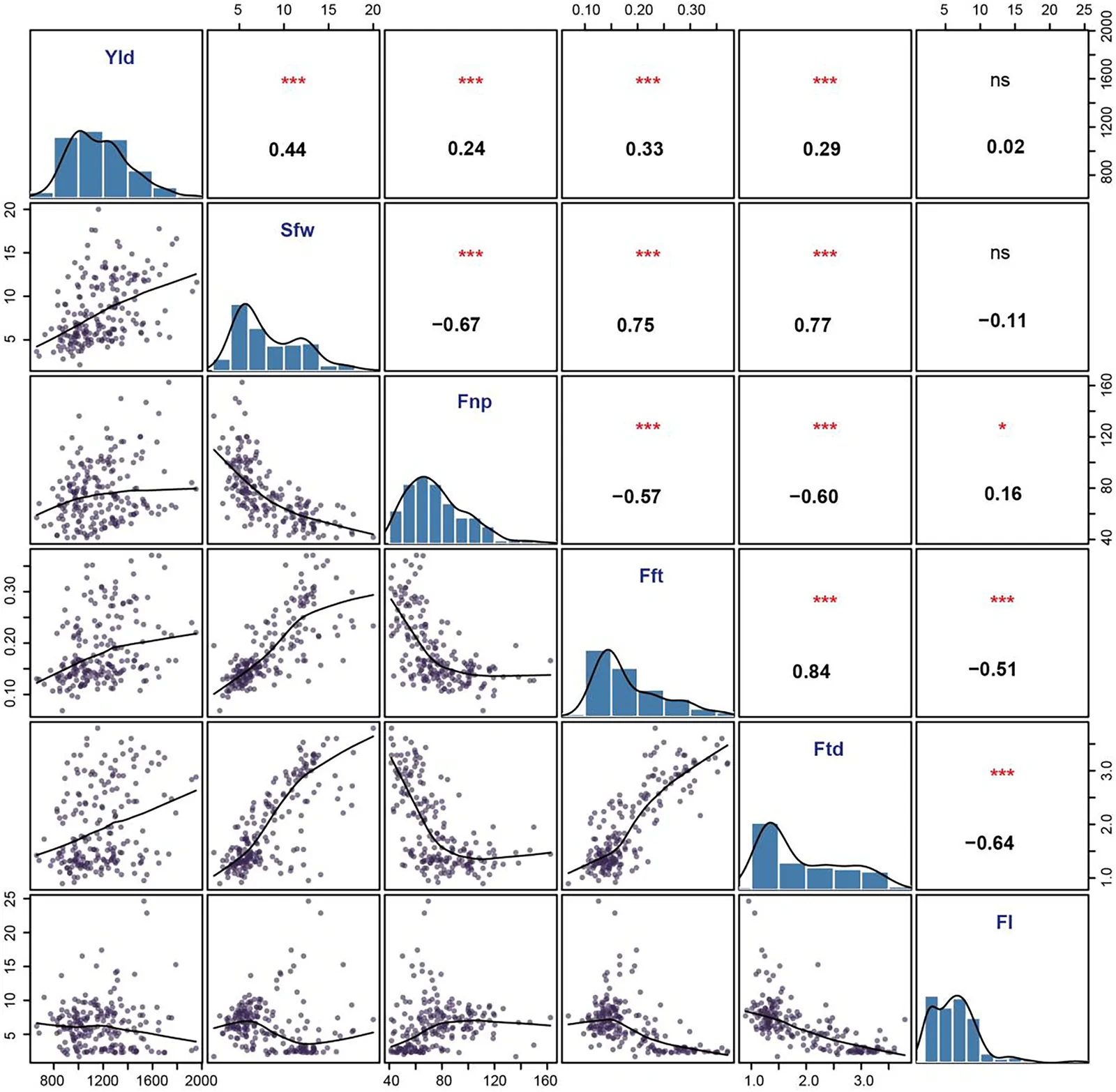
Correlation matrix analysis of yield and morphology traits. The matrix displays six variables: Yield (Yld), Single fruit weight (Sfw), Fruit number per plant (Fnp), Fruit flesh thickness (Fft), Fruit transverse diameter (Ftd), and Fruit length (Fl). The diagonal panels show the frequency distribution histograms and density curves for each trait. The lower triangular panels display pairwise scatter plots with fitted trend lines. The upper triangular panels show the Pearson correlation coefficients (*r*) and their corresponding significance levels (***P < 0.001; *P < 0.05; ns, not significant).

ANOVA revealed substantial genotypic variation across all investigated traits (**Supplementary Table S2**), with the largest proportion of variation observed for Yld (SS = 61,570,276.21). Environmental effects contributed more strongly to Yld and Fnp than to fruit morphology traits, including FL, Ftd, and Fft. Genotype × environment (G×E) interactions were also more pronounced for yield-related traits, whereas fruit morphology traits showed comparatively stable performance across environments (**Supplementary Table S2**). Medium to high broad-sense heritability (*H*²) was observed among these traits, ranging from 0.59 (Fft) to 0.97 (FL) (**Supplementary Table S3**). Fruit morphology traits overall exhibited higher heritability than yield-related traits, indicating they were less influenced by the environment and genotype × environment interactions.

### 3.3 GWAS identified significant loci associated with fruit morphology traits

Genome-wide association analysis was conducted to dissect genetic control of fruit morphology traits. A total of six significant loci associated with FL were identified, designated as *qFL1*-*qFL6* (**Figures 3A, B**; **Supplementary Figure 3**; **Table 2**). Among them, *qFL2* exhibited the strongest association signal, reaching a peak −log10(*P*-value) of 28.05, whereas *qFL5* represented another major locus with a peak value exceeding 14. Significant allelic effects were observed for all six loci. Accessions carrying the alternative (ALT) allele at *qFL1*, *qFL2*, *qFL3*, and *qFL6* showed significantly higher FL values than those carrying the reference (REF) allele (*P* = 2.1×10^-4^, 6×10^-6^, 5.9×10^-8^, and 2.2×10^-7^, respectively). In contrast, the ALT allele at *qFL4* and *qFL5* was associated with significantly reduced FL (*P* = 9.4×10–^6^ and < 2×10^-16^, respectively). Notably, *qFL5* displayed the largest phenotypic difference between allelic classes, suggesting that it is a major-effect locus contributing to natural variation in fruit length.

**Figure 3:**
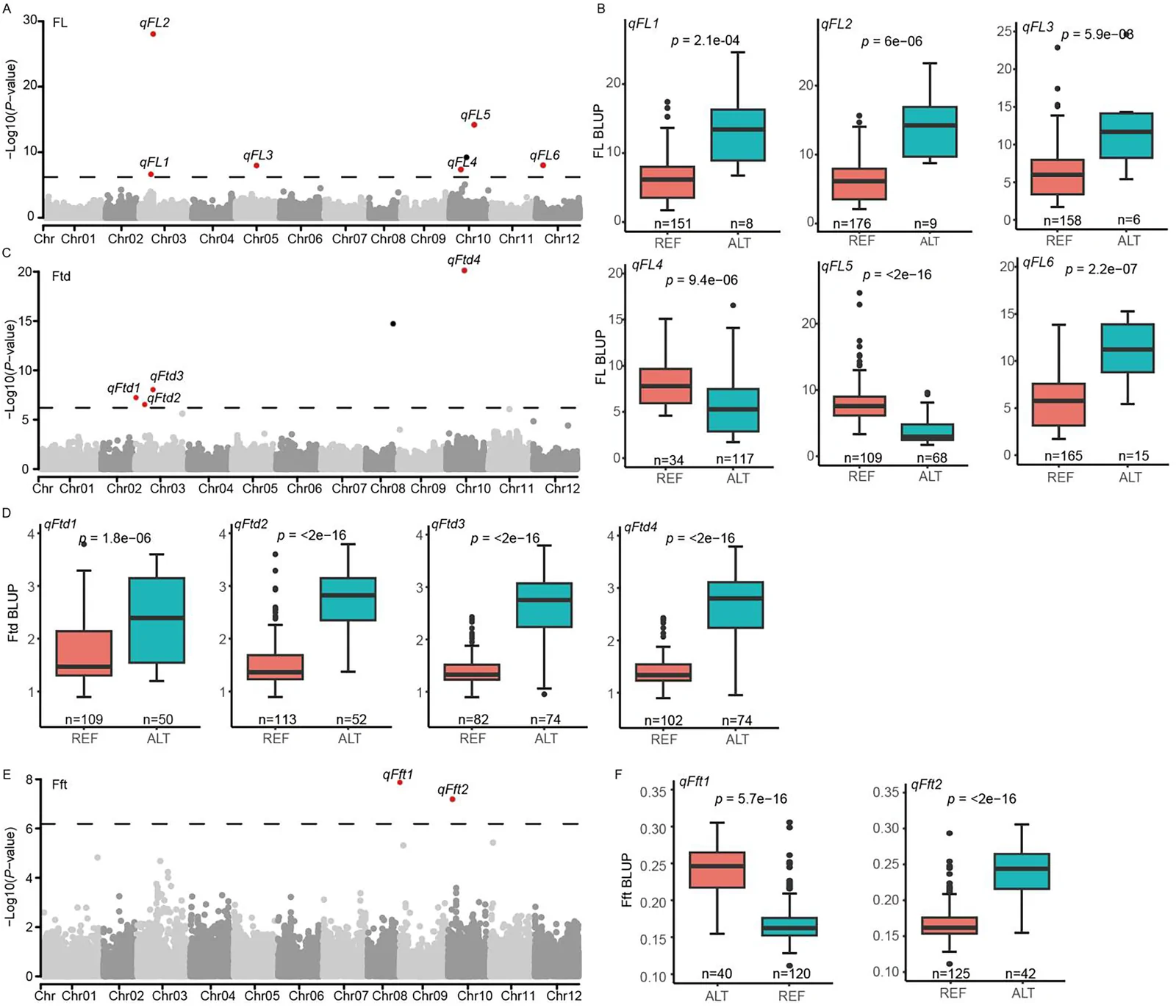
Genome-wide association mapping and allelic effects of loci associated with fruit morphology traits. Manhattan plots showing significant loci associated with fruit length [FL; **(A)**], fruit transverse diameter [Ftd; **(C)**], and fruit flesh thickness [Fft; **(E)**]. Significant loci are highlighted in red and labeled as *qFL1*-*qFL6*, *qFtd1*-*qFtd4*, and *qFft1*-*qFft2*, respectively. The horizontal dashed lines indicate the genome-wide significance thresholds. Boxplots show the allelic effects of the corresponding significant loci for FL **(B)**, Ftd **(D)**, and Fft **(F)**, based on best linear unbiased prediction (BLUP) values. REF and ALT represent accessions carrying the reference and alternative alleles, respectively. *P*-values were calculated using a two-sided Wilcoxon rank-sum test. The sample size (n) for each allelic group is indicated below the corresponding boxplot. In the boxplots, the center line represents the median, the box boundaries indicate the interquartile range (IQR), and the whiskers extend to 1.5 × IQR.

**Table 2:** Summary of GWAS associated loci identified for six traits.

Traits	locus	snp	REF	ALT	P_value	PVE(%)
Yld	*qYld*	Chr03-235149161	G	A	4.88E-07	12.21
Ftd	*qFtd1*	Chr03-3531791	G	T	5.79E-08	10.82
	*qFtd2*	Chr03-48699586	C	T	2.91E-07	7.18
	*qFtd3*	Chr03-92145818	A	C	8.98E-09	12.05
	*qFtd4*	Chr10-93850757	C	G	7.56E-21	33.24
Fnp	*qFnp1*	Chr03-47946253	G	A	9.70E-08	10.02
	*qFnp2*	Chr07-109771536	C	T	1.22E-08	12.10
	*qFnp3*	Chr08-153060734	C	G	1.09E-08	11.80
	*qFnp4*	Chr11-22673779	T	C	4.08E-07	8.78
	*qFnp5*	Chr11-176792206	C	T	3.59E-09	12.42
Fft	*qFft1*	Chr09-292789	A	C	1.35E-08	9.92
	*qFft2*	Chr10-22560227	C	G	6.51E-08	9.63
Sfw	*qSfw1*	Chr07-6407739	T	A	2.89E-10	15.04
	*qSfw2*	Chr07-217920275	G	A	3.57E-08	11.79
	*qSfw3*	Chr10-41894172	G	A	1.63E-07	10.38
	*qSfw4*	Chr11-148883023	C	T	3.99E-08	9.50
	*qSfw5*	Chr12-36112331	T	G	6.52E-09	12.82
FL	*qFL1*	Chr03-59239507	C	G	2.33E-07	9.33
	*qFL2*	Chr03-70913698	G	T	8.84E-29	44.24
	*qFL3*	Chr05-107913013	G	A	1.07E-08	10.94
	*qFL4*	Chr10-59945168	A	T	4.27E-08	11.43
	*qFL5*	Chr10-130071863	G	A	6.51E-15	21.93
	*qFL6*	Chr12-37799082	C	A	9.76E-09	11.48

For Ftd and Fft, four and two significant loci were identified, respectively (**Figures 3C–F**; **Supplementary Figure 3**; **Table 2**). Three Ftd-associated loci (*qFtd1*-*qFtd3*) were all located on chromosome 3, while *qFtd4* on chromosome 10 exhibited the strongest association signal. At all four loci, accessions carrying the ALT allele showed significantly increased Ftd values relative to REF allele carriers (*P* = 1.8×10–^6^ for *qFtd1* and P < 2×10–^16^ for *qFtd2*-*qFtd4*), indicating an association with greater fruit diameter. For Fft, two significant loci, *qFft1* and *qFft2*, were detected. The ALT allele at *qFft1* significantly decreased Fft (*P* = 5.7×10^-16^), whereas the ALT allele at *qFft2* significantly increased Fft (*P* < 2×10^-16^). Together, these loci were associated with natural variation in fruit morphology, including fruit elongation and radial growth.

### 3.4 GWAS identified significant loci associated with yield traits

To dissect the genetic basis governing yield-related traits, GWAS and subsequent phenotypic effect analysis were performed for the three yield-related traits, Fnp, Yld, and Sfw (**Supplementary Figure 3**). For Fnp, five significant quantitative trait loci (*qFnp1*-*qFnp5*) were identified across chromosomes 3, 7, 8, and 11, with *qFnp5* displaying the most prominent peak (**Figure 4A**; **Table 2**). Analysis of the BLUP values revealed different allele–trait associations across these loci: the ALT alleles at *qFnp1*, *qFnp2*, *qFnp3*, and *qFnp5* significantly increased fruit number (*P* < 1.4×10^-3^), whereas the ALT alleles at *qFnp4* contributed to a substantial reduction in Fnp (*P* = 0.01, **Figure 4B**).

**Figure 4:**
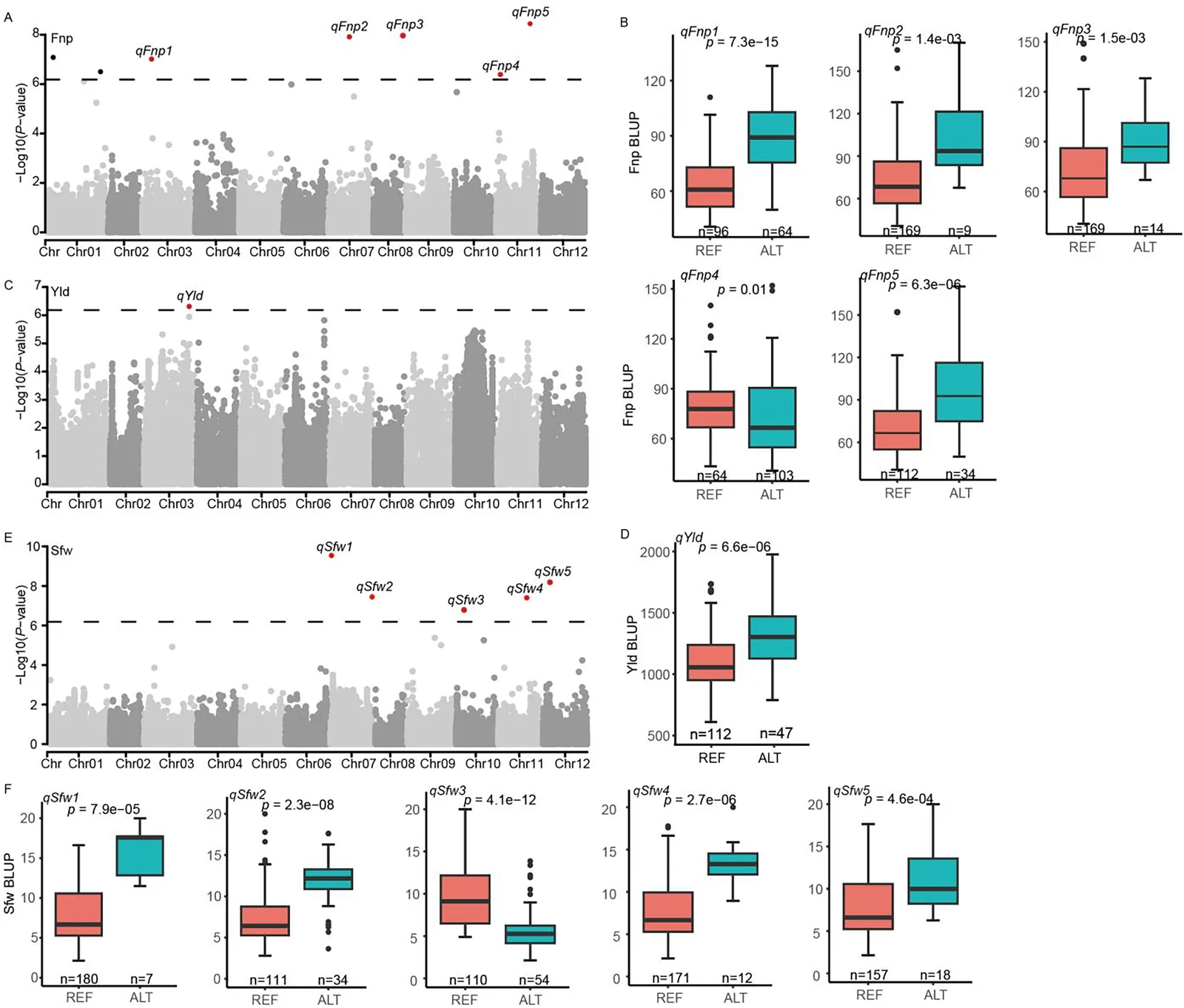
Genome-wide association studies and phenotypic effects of significant loci for yield traits. Manhattan plots illustrating the genomic regions significantly associated with fruit number per plant (Fnp) **(A)**, total yield (Yld) **(C)**, and single fruit weight (Sfw) **(E)** across the 12 chromosomes. The horizontal dashed lines represent the genome-wide significance threshold. Red dots indicate the peak SNPs for the identified significant quantitative trait loci (QTLs), labeled as *qFnp1*-*qFnp5*, *qSfw1*-*qSfw5*, and *qYld*, respectively. Boxplots displaying the phenotypic effect analysis based on Best Linear Unbiased Prediction (BLUP) values for the alleles at each identified locus. **(B)** BLUP values of Fnp for the reference (REF) and alternative (ALT) alleles at loci *qFnp1* to *qFnp5*. **(D)** BLUP values of Yld for the REF and ALT alleles at loci *qYld*. **(F)** BLUP values of Sfw for the REF and ALT alleles at loci *qSfw1* to *qSfw5*. Center lines represent the median; box limits indicate the 25th and 75th percentiles; whiskers extend to 1.5 times the interquartile range; points represent outliers. Sample sizes (n) for each allelic group and the statistical significance (*P*-values) determined by a two-sided Wilcoxon rank-sum test are indicated at the bottom and top of each boxplot, respectively.

Concurrently, significant loci were identified for Yld and Sfw. Regarding Yld, a significant locus (*qYld*) was detected on chromosome 3 (**Figure 4C**). Accessions carrying the ALT allele at *qYld* had a higher Yld BLUP value than REF-allele carriers in this panel (**Figure 4D**). Five distinct loci (*qSfw1*-*qSfw5*) were pinpointed for Sfw, among which *qSfw1* on chromosome 7 exhibited the highest statistical significance (**Figure 4E**). Notably, the ALT alleles at *qSfw1*, *qSfw2*, *qSfw4*, and *qSfw5* loci were associated with greater single fruit weight than the corresponding REF alleles (*P* < 4.6×10^-4^, **Figure 4F**), suggesting that they may contribute to increased Sfw.

### 3.5 Linkage disequilibrium and candidate genes within major loci

The maximum mean short-range LD was *r*^2^ = 0.6608, giving a half-maximum threshold of *r*^2^ = 0.3304. The weighted LOESS curve crossed this threshold at 414 kb (**Figure 5**). Based on the rounded 420-kb LD half-decay distance, each GWAS-associated loci was defined as the lead SNP plus 420 kb on either side. Across the 23 GWAS-associated loci, 320 QTL-gene associations representing 318 unique genes were identified (**Supplementary Table S4**).

**Figure 5:**
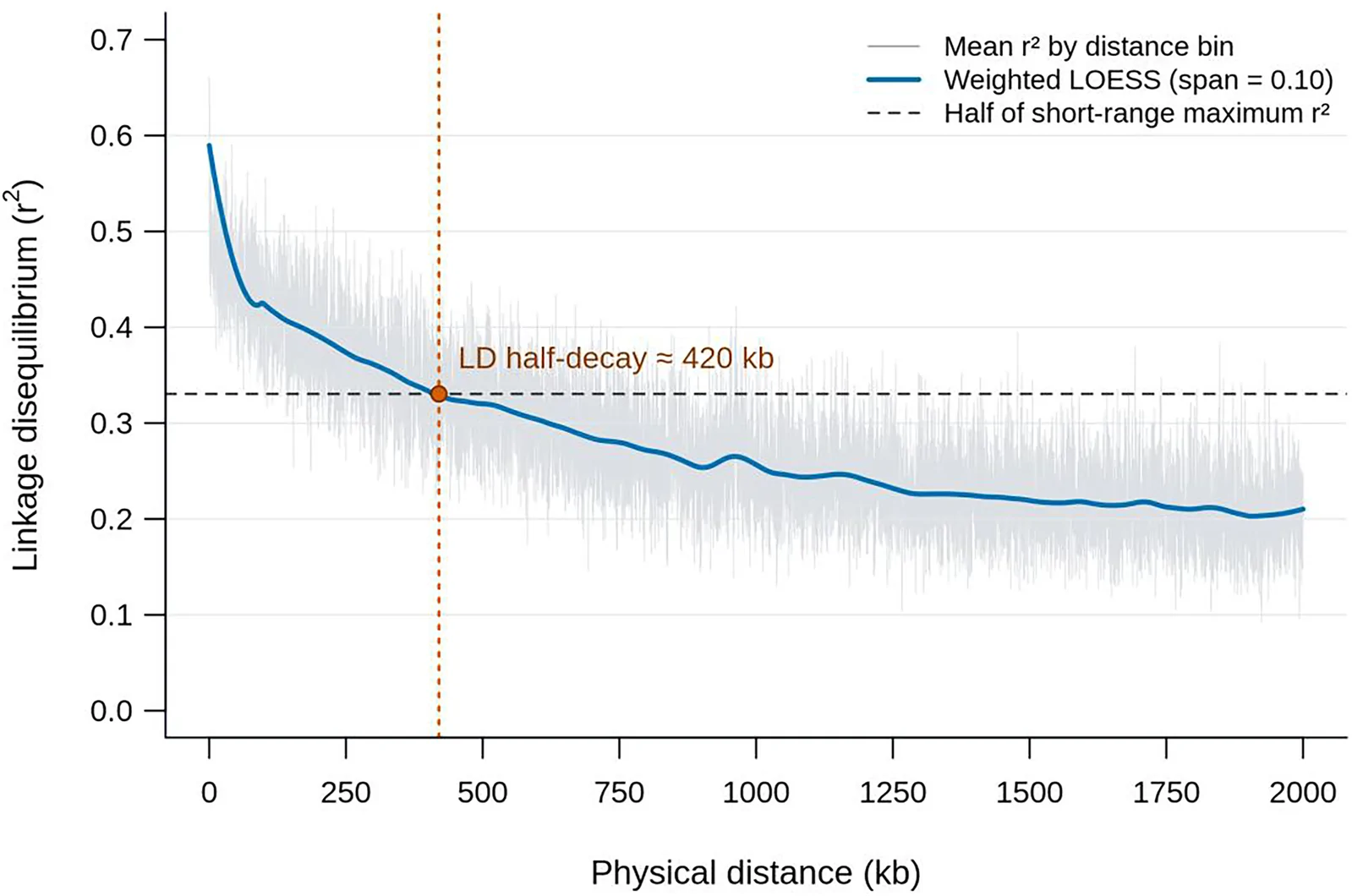
Genome-wide linkage disequilibrium decay in the 195-accession pepper association panel. The gray line shows mean pairwise *r*^2^ by physical-distance bin, and the blue line shows the weighted LOESS fit. The horizontal dashed line marks half of the maximum mean *r*^2^ observed within 1 kb (*r*^2^ = 0.33). The orange dashed line indicates the rounded LD half-decay distance of 420 kb used to define QTL candidate intervals.

Several major loci contained genes with functions relevant to fruit development or yield formation. The *qFL2* interval contained only two annotated genes, including *LOC107863364*, which encodes gibberellin 20 oxidase 1 (GA20ox1) and lies approximately 98 kb from the lead SNP. *CaGA20ox1* is involved in gibberellin biosynthesis and has previously been implicated in pepper fruit development and elongation (Tsaballa et al., 2012). The *qFL5* interval contained *LOC107845902*, annotated as an OVATE family transcriptional repressor, approximately 88 kb from the lead SNP. This gene is physically coincident with the previously characterized *CaOFP20* region on pepper chromosome 10, for which functional silencing and genetic analyses demonstrated a role in suppressing fruit elongation (Borovsky et al., 2022). The *qYld* interval contained *LOC107864101*, a putative candidate approximately 36 kb from the lead SNP and annotated as the chloroplastic photosystem II D1 precursor-processing protein PSB27-H2, together with genes encoding a WNK9-like protein kinase, PAF1, and SFH4. Arabidopsis *PSB27-H2/LPA19* participates in D1 precursor processing and photosystem II biogenesis (Wei et al., 2010). Other plausible genes included a polygalacturonase in *qFft1* and a *bHLH089* transcription factor in *qFnp5*. Polygalacturonases have established roles in pepper fruit cell-wall disassembly, softening, and abscission, although a direct effect on flesh thickness has not been demonstrated (Rao and Paran, 2003).

## 4 Discussion

Fruit morphology and yield are among the most important agronomic traits in pepper breeding because they directly influence market preference, production efficiency, and economic value (Paran and van der Knaap, 2007). Dissecting their genetic architecture is therefore essential for accelerating molecular breeding and germplasm improvement. In the present study, substantial phenotypic variation was observed for all investigated traits, with coefficients of variation ranging from 40.43% to 57.09%. Such extensive variation may reflect the diverse genetic backgrounds of the accessions and their responses to different environmental and breeding conditions. Moreover, all traits exhibited continuous distributions, consistent with polygenic inheritance. These findings indicate that the Guizhou pepper collection harbors abundant genetic diversity and represents a valuable resource for identifying loci controlling complex agronomic traits.

Population structure analysis further revealed substantial genetic differentiation within the collection. ADMIXTURE, PCA, and phylogenetic analyses consistently divided the accessions into eight genetic subgroups, while several accessions displayed admixed ancestry. These results indicate complex genetic backgrounds within the Guizhou pepper collection, although the underlying demographic and breeding processes require further investigation (Feng et al., 2020). Population stratification is a major source of false-positive associations in GWAS; therefore, accurate characterization of population structure is essential for reliable locus detection (Price et al., 2006). Combined with the high-density SNP dataset generated by SLAF-seq, the observed population structure provided sufficient statistical power and genomic resolution for identifying trait-associated loci. The relatively uniform distribution of more than 76,000 high-quality SNP markers across the pepper genome further increased the reliability of the association signals detected in this study.

Comparison with previous pepper genetic maps revealed both recurrent genomic patterns and potentially population-specific signals. Classical studies identified major fruit size and shape loci on chromosomes 2, 3, 4, and 10, while subsequent high-density mapping detected a concentration of fruit-related QTLs on chromosome 3 (Rao et al., 2003; Zygier et al., 2005; Han et al., 2016). In the present panel, *qFL1* and *qFL2*, together with three of the four Ftd loci, were also located on chromosome 3. At the chromosome level, these results agree with the reported importance of the *fs3.1* region and with the fruit-length and fruit-width QTLs identified by Lee et al. (2020). The location of *qFL3* on chromosome 5 likewise agrees at the chromosome level with a fruit-length QTL detected in the Perennial × Dempsey population (Lee et al., 2020). Chromosome 10 was also repeatedly represented in our results through *qFL4*, *qFL5*, *qFtd4*, *qFft2*, and *qSfw3*, consistent with previous fruit-morphology associations reported on this chromosome (Colonna et al., 2019; Borovsky et al., 2022). Nevertheless, because these studies used different populations, marker systems, and reference-genome assemblies, chromosome-level agreement cannot be interpreted as definitive physical colocalization. In contrast, *qFft1* and *qFft2*, located on chromosomes 9 and 10, respectively, differ from previously reported pericarp-thickness loci on chromosomes 2, 4, 6, 7, 11, and 12 (Dwivedi et al., 2015; Lee et al., 2020). These differences may reflect allelic heterogeneity in the Guizhou germplasm, differences in phenotyping and statistical power, or the environmental sensitivity of fruit flesh thickness.

Candidate-gene evidence was strongest for the two major Fl loci, although the level of support differed between them. The *qFL2* interval contained *LOC107863364*, predicted to encode gibberellin 20-oxidase 1 (GA20ox1), approximately 98 kb from the lead SNP. Expression of *CaGA20ox1* differs between long- and round-fruited pepper cultivars and responds to manipulation of *CaOvate*, implicating gibberellin biosynthesis in pepper fruit elongation (Tsaballa et al., 2012). Together with its location within the associated interval, this evidence highlights the GA20-oxidase gene *LOC107863364* as a strong candidate for fruit-length variation in our population. By contrast, *qFL5* provides a direct connection with previous pepper genetics. *LOC107845902* is located approximately 88 kb from the lead SNP at approximately 130.16 Mb on chromosome 10 of the Zunla-1 assembly and is physically coincident with the previously characterized *CaOFP20* region within the *fs10* interval. Silencing of *CaOFP20* increased fruit elongation in two pepper genetic backgrounds, and natural variation in this gene was associated with fruit shape (Borovsky et al., 2022). The physical colocalization, pronounced allelic effect observed in the present panel, and previous functional evidence collectively identify *LOC107845902*/*CaOFP20* as the highest-priority candidate in this study.

Yield-component loci showed more limited but still informative agreement with previous studies. *qSfw1* and *qSfw2* were located on chromosome 7, on which Lee et al. (2020) identified the stable major fruit-weight QTL *PD_FWg7.1*. Additional *qSfw* loci on chromosomes 10, 11, and 12 occurred on chromosomes that also contained fruit-weight associations in that multi-year study. Because the physical coordinates were obtained using different pepper genome assemblies, these observations represent chromosome-level replication rather than confirmed colocalization. Fnp and Yld have received less genetic investigation than fruit size and shape, and the *qFnp* and *qYld* loci may therefore represent useful population-specific targets. Within *qYld*, *LOC107864101*, located approximately 36 kb from the lead SNP, is predicted to encode the photosystem II D1 precursor-processing protein PSB27-H2. Its *Arabidopsis* homolog *LPA19* facilitates D1 precursor processing during photosystem II biogenesis, and *lpa19* mutants exhibit reduced photosystem II accumulation and photochemical efficiency (Wei et al., 2010). These findings suggest a possible connection between photosynthetic performance and plant productivity. The *WNK9-like protein kinase*, *PAF1*, and *SFH4* genes within the same interval therefore remain alternative candidates.

## 5 Conclusion

This study characterized the phenotypic and genetic diversity of 195 pepper accessions from Guizhou and identified 23 loci associated with six fruit morphology and yield-related traits. Genome-wide LD declined to half of its short-range maximum at approximately 414 kb, supporting the use of 420-kb flanking regions for candidate-gene identification. These intervals contained 318 unique genes. Comparison with previous studies indicated recurrent contributions of chromosomes 3, 7, and 10 to pepper fruit morphology and weight. Among the prioritized genes, *LOC107845902*/*CaOFP20* within *qFL5* was the strongest candidate because of its physical correspondence with the functionally characterized *fs10* region, whereas *LOC107863364*/*GA20ox1* within *qFL2* represents a biologically plausible but unvalidated candidate. Genes identified within *qYld* and other loci remain preliminary hypotheses requiring further investigation. Overall, these association loci and allele-trait relationships provide hypotheses for marker development, although multi-environment validation, fine mapping, expression analysis, and functional experiments will be necessary before their application in molecular breeding.

## Data Availability

The genotype can be accessed at Figshare [https://doi.org/10.6084/m9.figshare.34516479].
